# Progress in State Medicine

**Published:** 1902-12-06

**Authors:** 


### PROGRESS IN STATE MEDICINE.

(*Concluded from page* 145.)

THE Sanitary Committee[5] of the East Riding of Yorkshire County Council have directed that copies of a report on Tuberculosis drawn up by their medical officer, Mitchell-Wilson, shall be sent to the Urban and Rural District Councils, with the request that their medical officers of health shall consider whether it is desirable that voluntary notification of tuberculous disease should be adopted in their respective districts. The report deals chiefly with the following seven registration districts in the East Riding:—Pocklington, Howden, Beverley, Patrington, Skirlaugh, Driffield and Bridlington. It is pointed out that when the death-rates from phthisis during the ten years, 1891-1901, are compared with those of 1871-1880, they show that there has been a reduction throughout the whole of England and Wales of 34 per cent. In the whole of the East Riding districts the reduction was 34 per cent.; but in the seven chosen registration districts the reduction was 38 per cent. The death rate from other tuberculous diseases has also been lessened in the same period, but to a far less extent, viz, 16 per cent. in England and Wales, 10 per cent. for the whole of the East Riding, and 16 per cent. for the seven registration districts. When the several death-rates in these districts are calculated on the population of each, it is found that if the average returns for the last ten years are taken on the same basis, throughout the whole of the administrative county there were every year 145 deaths from phthisis, and 58 by other forms of tubercular disease. A large majority of the victims of this disease are persons in the prime of life, between the fifteenth and thirty-fifth years. In addition to the 58 deaths annually caused by other forms of tuberculous disease, there are at least twice that number always ill from these, the majority of them being children and young persons. In drawing attention to the great dangers caused by promiscuous expectoration, he emphasises the fact that the risk of infection is naturally greatest in badly ventilated and crowded dwelling-houses and workshops, as well as in public buildings and conveyances. Past results are an encouragement to persevere with all sanitary work which will bring about more thorough drainage of the subsoil, and which endeavours to provide houses with more air-spaces and good thorough ventilation. Wherever a death has been registered from phthisis, especially if in an unhealthy or crowded dwelling, the sanitary authorities should offer to have the clothing and premises disinfected. The same authorities should encourage voluntary notification, and follow the example already set in some places, in making arrangements to have the sputa of doubtful cases tested. It is not yet satisfactorily decided if the powers given to County Councils under the Isolation Acts of 1893 and 1901, to assist in providing or maintaining hospitals for the reception of patients suffering from infectious diseases, apply to institutions set apart for persons suffering from consumption. Bullock[6] remarks that up to the present time, no chemical substance was known that rapidly disinfected sputum and at the same time did it effectively. The resistance of the bacillus to light was very slight, and death took place in a few minutes or hours. Exposure to cold was well borne by the bacillus. Saturated moist heat was most potent, and in institutions containing phthisical patients, this method was "par excellence" for the disposal of sputum. The sputum was discharged from the mouth of the individual affected in the form of a spray, which was contained of droplets. The droplets varied very much in different cases. Some individuals were found to eject the spray very much further than others. It had been shown that loud speaking or coughing could carry the spray from the mouth. The duration of the life of the droplet was most important. Thirty minutes after a patient had coughed into the receptacle it was found that the air was infectious. With certain currents of air it was found that the droplets could be kept suspended in the atmosphere. The present forms of pocket spittoons were not satisfactory. Paper handkerchiefs, to be burnt after use, were preferable. It might be doubted whether anyone knew of a completely satisfactory method of disinfecting rooms. There should be spittoons in hospitals, railway stations, and such places, and arrangements made for their proper disinfection. What was urgently needed was the provision of sanatoria where advanced cases could go to die. Pattin[7] considers that pulmonary tuberculosis is the primary lesion much more rarely than is commonly assumed to be the case. He believes this to be true of those who die of phthisis. Phthisis is rather evidence of the development of the disease than of its invasion. Too much attention was paid at the recent British Congress to the pulmonary type of tuberculosis, and an insufficient amount to other types. Voluntary notification as compared with compulsory amounts at most to a possibility of obtaining a knowledge of some of the houses in which there are cases of pulmonary tuberculosis, but promises only a very ineffective semblance of control. In his own district 95 per cent. of the dwellings in which deaths from phthisis took place last year were disinfected through knowledge gained from the weekly returns of the district registrars to the medical officer of health. The latter can inform himself of the existence of tuberculous disease in dwellings by desiring his district inspectors to inquire upon their house-to-house visits if there is anyone suffering from "consumption" in the dwellings, and communicating the fact to him. Further, useful information might be collected from the officials of the union infirmary and from the house physicians of the public hospitals. As a preventive measure, he advocates that no consumptive person should be permitted to land from a ship without notification of his destination being sent to the medical officer of health by the ship's doctor. Similar notification should be required from the friends when known consumptives proposed to stay at health resorts, so that the infected vacated house might be disinfected. To the lay mind an infectious disease which is notified in accordance with an Act of Parliament is alone really serious. In Glasgow[8] and Liverpool the corporation tramway companies have made spitting in the tramways a punishable offence. The County Council of Glamorganshire, wishing to prohibit spitting in public places by by-law, have been informed by the Secretary of State that, unless any objections are lodged, he will allow the by-law to come into force if its operation is confined to public carriages, public waiting-rooms, public halls, and places of public entertainment. He thinks that the by law cannot properly be made to apply to churches, chapels, schools, and shops.

[5] Sanit. Rec., May 15, 1902. [6] Sanit. Rec., May 29, 1902. [7] Brit. Med. Jour., June 7, 1902. [8] Brit. Med. Jour., Aug. 2, 1902.

